# Facile Construction of Bio-Based Supramolecular Hydrogels from Dehydroabietic Acid with a Tricyclic Hydrophenanthrene Skeleton and Stabilized Gel Emulsions

**DOI:** 10.3390/molecules26216526

**Published:** 2021-10-28

**Authors:** Caiyun Lin, Yuying Li, Weishan Tang, Shufeng Zhou, Xiaoping Rao

**Affiliations:** College of Chemical Engineering, Huaqiao University, Xiamen 361021, China; Lcy10286@163.com (C.L.); lyy_0128@126.com (Y.L.); 18815610689@163.com (W.T.); szhou@hqu.edu.cn (S.Z.)

**Keywords:** bio-based, supramolecular hydrogels, dehydroabietic acid, oil-in-water, gel emulsions

## Abstract

Supramolecular hydrogels have attracted great attention due to their special properties. In this research, bio-based supramolecular hydrogels were conveniently constructed by heating and ultrasounding two components of dehydroabietic acid with a rigid tricyclic hydrophenanthrene skeleton and morpholine. The microstructures and properties of hydrogels were investigated by DSC, rheology, SAXS, CD spectroscopy, and cryo-TEM, respectively. The critical gel concentration (CGC) of the hydrogel was 0.3 mol·L^−1^ and the gel temperature was 115 °C. In addition, the hydrogel showed good stability and mechanical properties according to rheology results. Cryo-TEM images reveal that the microstructure of hydrogel is fibrous meshes; its corresponding mechanism has been studied using FT-IR spectra. Additionally, oil-in-water gel emulsions were prepared by the hydrogel at a concentration above its CGC, and the oil mass fraction of the oil-in-water gel emulsions could be freely adjusted between 5% and 70%. This work provides a convenient way to prepare bio-based supramolecular hydrogels and provides a new method for the application of rosin.

## 1. Introduction

Supramolecular gels are a typical class of physical gels that integrate three-dimensional network structures through intermolecular hydrogen bonding, π-π stacking, van der Waals forces, electrostatic forces, coordination, and weak dipole–dipole interactions [1,2]. This relies on interfacial tension and capillary interaction to make the solvent present therein lose its mobility and form a viscoelastic soft solid material [3]. The difference between supramolecular gels and conventional covalent bond polymer gels is their non-covalent bond self-assembly. The gelation of supramolecular gels is a multi-stage, self-assembly process of gel factors that are driven by non-covalent bonds to form aggregates of smaller size and continue to self-assemble between aggregates to generate nanofibrous structures. The nanofibers are intertwined to form a three-dimensional mesh structure, which then wraps around a large amount of solvent to form macroscopically visible gels [4]. Supramolecular gels tend to have good phase change reversibility. In other words, the phase change process between the gel-sol can be controlled by heating, chemicals, ultrasound, light, electricity, oxidation/reduction, and shear stimulation, etc. [5,6,7,8,9]. These properties mean that such gels exhibit potential applications in sensing [10,11], controlled drug release [12,13,14], removal of toxic dyes [6,15,16,17], and micro- and nanomaterial preparation [18,19].

Supramolecular hydrogels have attracted widespread interest, and the key factors to form hydrogels are the structures of gelators. Natural products are often used to design supermolecule hydrogels, which have the following advantages: (1) bio-based supramolecular hydrogels not only form low-molecular-weight hydrogels as drug delivery systems, but also have synergistic therapeutic effects on diseases due to their pharmacological activity; (2) due to the inherent advantages of natural products, bio-based supramolecular hydrogels have good safety, biodegradability safety, biodegradability, biocompatibility, biostability, and low toxicity [20,21,22]. Nowadays, the main raw materials for the preparation of supramolecular hydrogels are amino acids [23,24], alginic acid [25,26], and bile acids [19,27,28,29,30], etc. In general, rigid hydrophobic groups are essential for the synthesis of gel factors. Bile acid contains a rigid skeletal structure of pentacyclic triterpene, which is an important raw material for the preparation of supramolecular gels. However, bile acids are mainly extracted from the bile of animals, which is a complicated extraction method and provides limited resources.

Rosin is an important renewable natural resource; the main components of rosin are resin acids, which have a rigid tricyclic hydrophenanthrene skeleton structure and monocarboxylic acid. Rosin is obtained from the resin of pine trees, which can obtain a large amount of raw material compared with other natural products by solvent extraction through thin-layer column separation. Rosin acids are also biocompatible FDA-approved food additives in chewing gum and soft drinks. The introduction of rosin acid into the structure of hydrogel gelators is expected to obtain a series of hydrogels with favorable biocompatibility and biodegradability, complying with green chemistry requirements. The tricyclic hydrophenanthrene characteristics (20 carbon atoms) of rosin acids have strong hydrophobicity, although they are weaker than those of triterpenoid cholesterol that contains 30 carbon atoms [31]. It can form gelators after introducing another hydrophobic portion into rosin [31,32]. Some rosin-based surfactants can form hydrogel combined with molecules or counterion. Cui reported that supramolecular hydrogels with chiral nanofibril structures are formed from β-cyclodextrin and a rosin-based amino acid surfactant [1]. Song also reported that an amphiphilic hydrogelator was derived from dehydroabietic acid [31] with an oxidized amine structure. However, studies and reports on surfactant-based supramolecular hydrogels need complex synthesis procedures. 

Gel emulsions, which combine the characteristics of both emulsions and gels, have caused a lot of attention in recent years [33]. Similar to conventional emulsions, gel emulsions are also two-phase systems in which one phase is dispersed as droplets in the other phase, while they also exhibit physical gel behavior [1]. As a result, gel emulsions have a unique rheological behaviour and mesoscopic structure, which is why they have a wide range of applications in the preparation of food, cosmetics, drug delivery, tissue engineering, and advanced porous materials, among many others [34,35,36]. Due to the tight buildup of dispersed droplets, heavy ionic liquids are usually very elastic and have the advantages of high stability, high ability to carry oil or water, and good resistance to external forces [37,38,39,40]. Herein, rosin-based supramolecular hydrogels were prepared using dehydroabietic acid and morpholine as raw materials. The morphology of the micron-scale structures and the properties of the supramolecular hydrogels were studied. Meanwhile, oil-in-water gel emulsions were also developed. This work provides a new strategy to construct supramolecular hydrogel and exploit new applications of natural products.

## 2. Results and Discussion

### 2.1. Phase Behavior of Supramolecular Hydrogels

**Figure 1:**
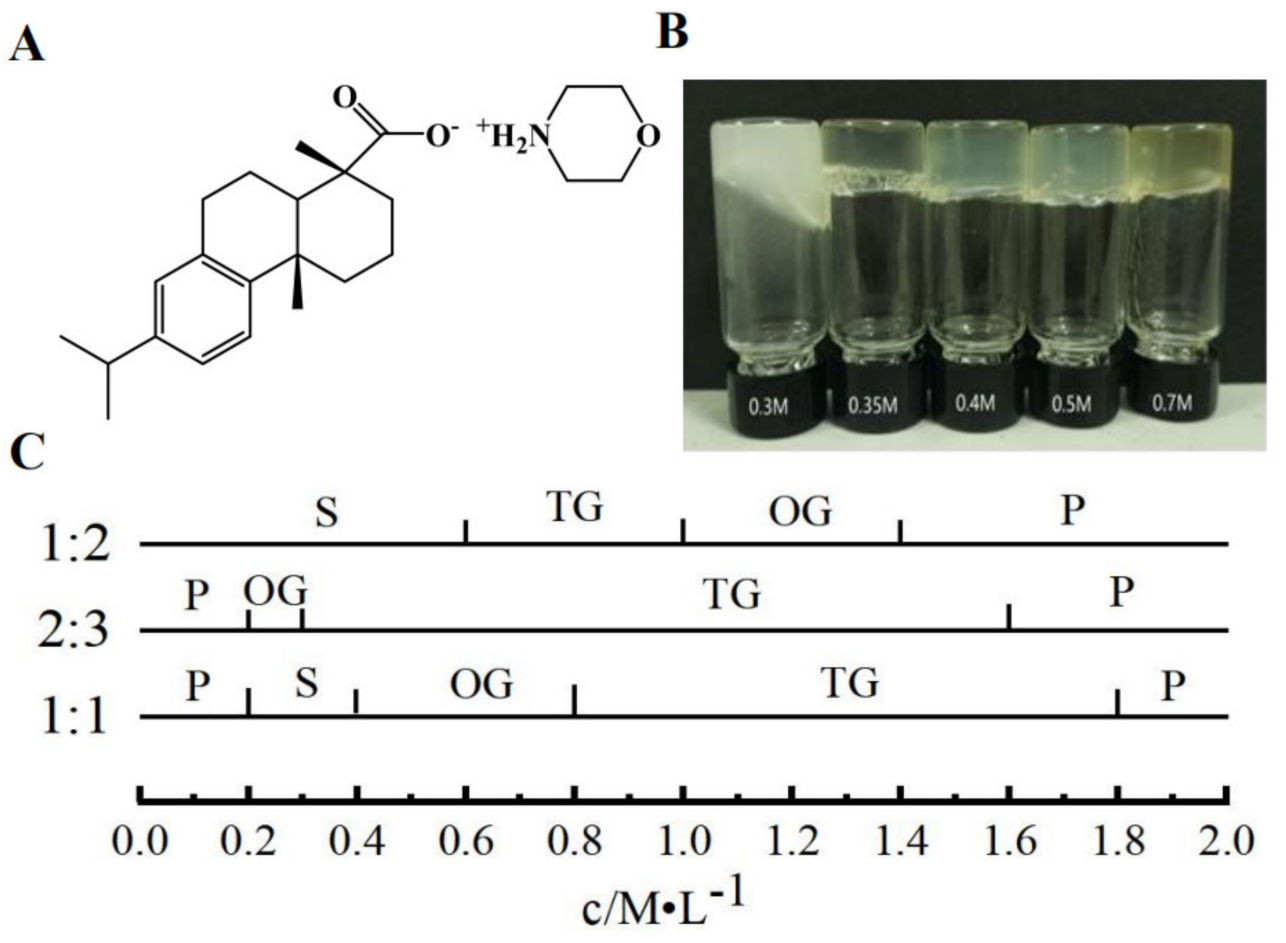
(**A**) Molecular structure of gelator; (**B**) appearance of hydrogels of different concentrations; (**C**) Phase diagrams for different molar ratios of dehydroabietic acid to morpholine. Key: P, precipitate; S, solution; OG, opaque gel; TG, transparent gel.

Gelator plays an important part in the formation of supramolecular hydrogel; dehydroabietic acid can form a transparent gel with morpholine. In contrast to other gel preparation processes, dehydroabietic acid morpholine salt can be prepared by simply heating and sonicating a gelator, leading to gelation in the water solution. The preparation method is simple and the raw material is dehydroabietic acid, which is isolated from disproportionated rosin, a renewable green resource which is an FDA-approved food additive. Morpholine is also used as a raw material to produce fatty acid morpholine salt used as fruits coating. Figure 1C shows simple phase diagrams of the different molar ratios of dehydroabietic acid and morpholine at different concentrations. A large range of gels can be formed when the molar ratio of dehydroabietic acid to morpholine is 2:3. The concentration range of transparent hydrogels becomes narrower when the molar ratio of dehydroabietic acid to morpholine is 1:1 or 1:2. We have studied the 2:3 dehydroabietic acid to morpholine system in detail to assess the detailed properties of the hydrogels. As shown in Figure 1B, we prepared supramolecular hydrogels with different concentrations of dehydroabietic acid with morpholine. It can be noted that the CGC of the supramolecular hydrogel is 0.3 M. The CGC of this hydrogel is still relatively large compared to other macromolecular gels, which are to be further refined.

### 2.2. DSC Analysis of the Supramolecular Gels

**Figure 2:**
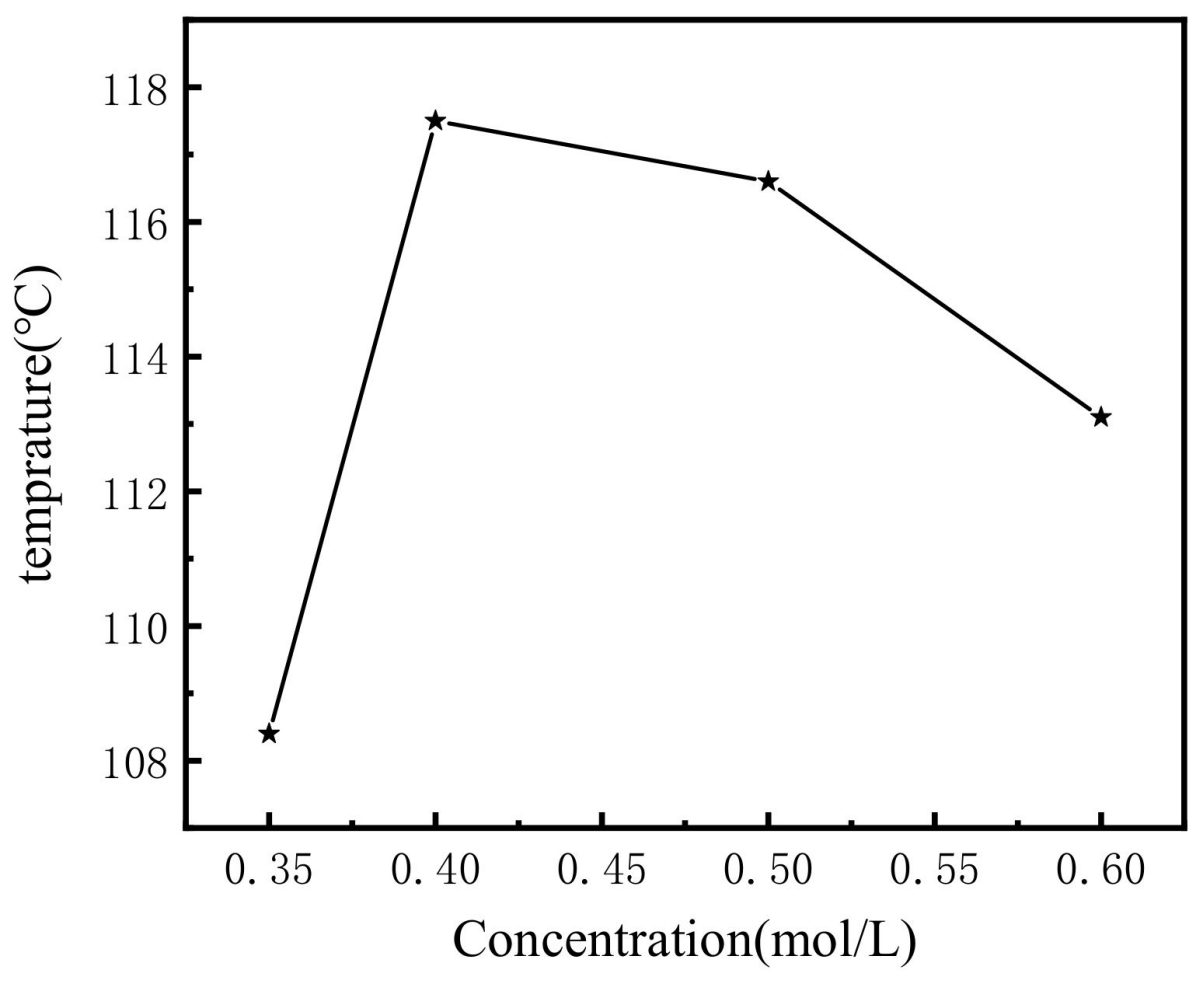
Plot of T_gel_ of with different concentrations.

The gel temperatures (T_gel_) of the hydrogel with different gelator concentrations of 0.35, 0.4, 0.5, and 0.6 M were 108, 117, 116, and 113 °C, respectively, and the results are shown in Figure 2. The gel temperature increased at first, and then decreased with increasing gelator concentration. It was also demonstrated that the supramolecular hydrogel had a higher gel temperature than the general supramolecular hydrogel, indicating a better gelation performance. The currently reported gelation temperatures of supramolecular gels range from 40–90 °C, and the gelation temperature of supramolecular hydrogels depends mainly on the structure and concentration of the gel factor itself, as well as on the influence of the solvent [27,41,42,43]. In contrast, the gelation temperature of the supramolecular gels measured by DSC in this paper was greater than 100 °C, indicating that the hydrogels have better stability. The rigid backbone structure of the gel factor and the small number of hydrophilic groups may be an important reason for the high gelation temperature. 

### 2.3. Rheological Properties of the Supramolecular Gels

**Figure 3:**
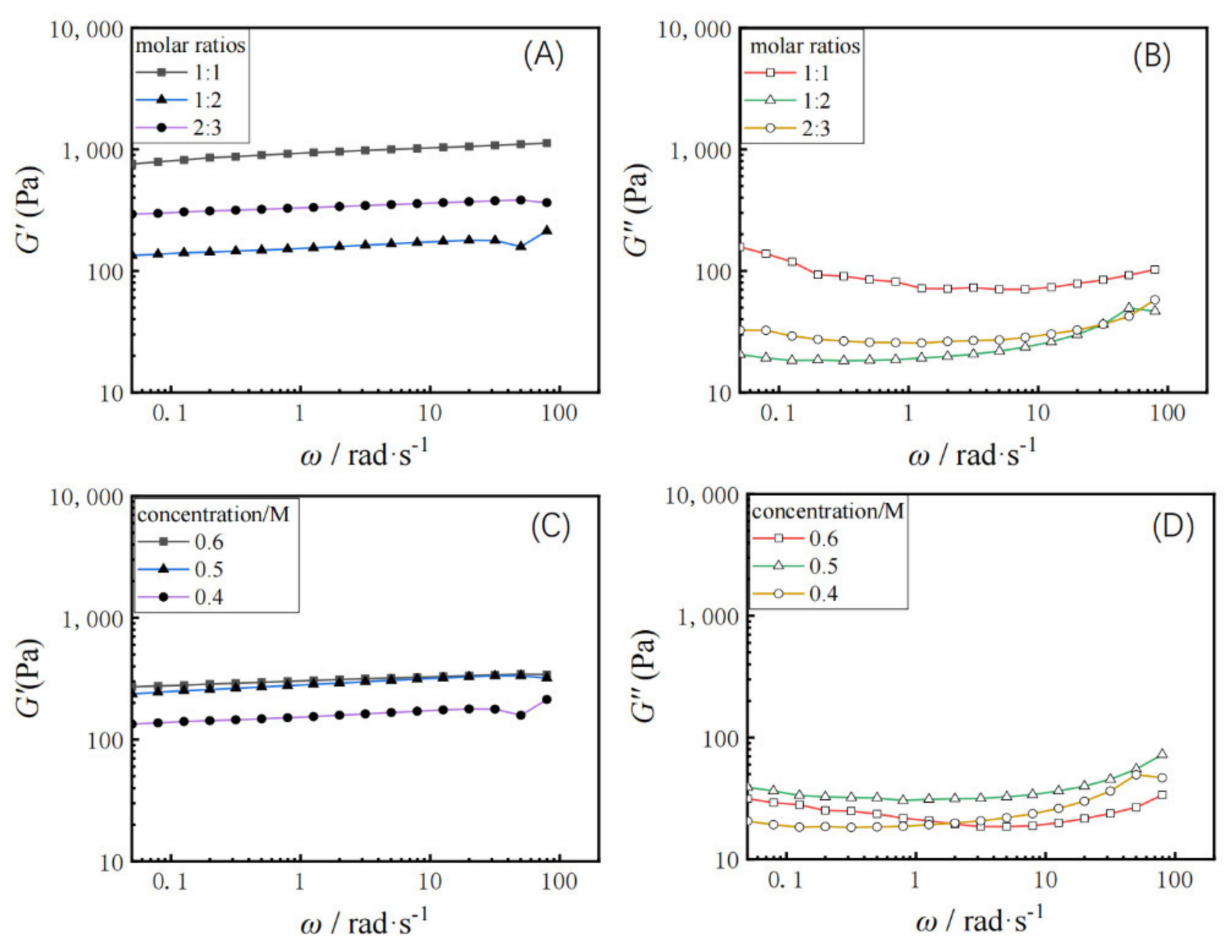
Variation of elastic modulus G′ and viscous modulus G” with shear frequency (ω) at 25 °C for hydrogels with different molar ratios (**A**,**B**) and with different concentration (**C**,**D**).

Dynamic rheology was measured for different molar ratios of dehydroabietic acid to morpholine at 1:1, 1:2, and 2:3, respectively. The elastic modulus (G’) of the hydrogel and the viscous modulus (G”) with change of angular frequency (ω) was tested. As shown in Figure 3A,B, the elastic modulus (G’) of the hydrogel is greater than the viscous modulus (G”), which is consistent with the properties of the gel. The elastic modulus G’ and viscous modulus G’’ of the hydrogel characterizes the mechanical properties of the hydrogel [44,45]. From the data, it is clear that the dehydroabietic acid morpholine supramolecular hydrogel has very good mechanical properties to the order of 10^3^ Pa. This may be strongly related to the rigid skeletal structure of the tricyclic hydrophenanthrene skeleton of rosin. Furthermore, G’ and G” decreased and then increased with the increasing molar ratio of dehydroabietic acid to morpholine.

Figure 3C,D shows the rheological properties of the hydrogel samples formed at gelator concentrations of 0.4, 0.5, and 0.6 M, respectively. The G’ values for all gels samples were always higher than the G” values for the range of oscillation frequencies studied, again indicating a typical gel-like behaviour. It was also found that at fixed frequencies, the value of G’ increased slightly with increasing concentration of gelators, indicating increased elasticity of the gel at higher concentrations, while G’’ showed an increase first and then a decrease with gelator concentration increment.

### 2.4. Cryo-TEM of Supramolecular Hydrogel

**Figure 4:**
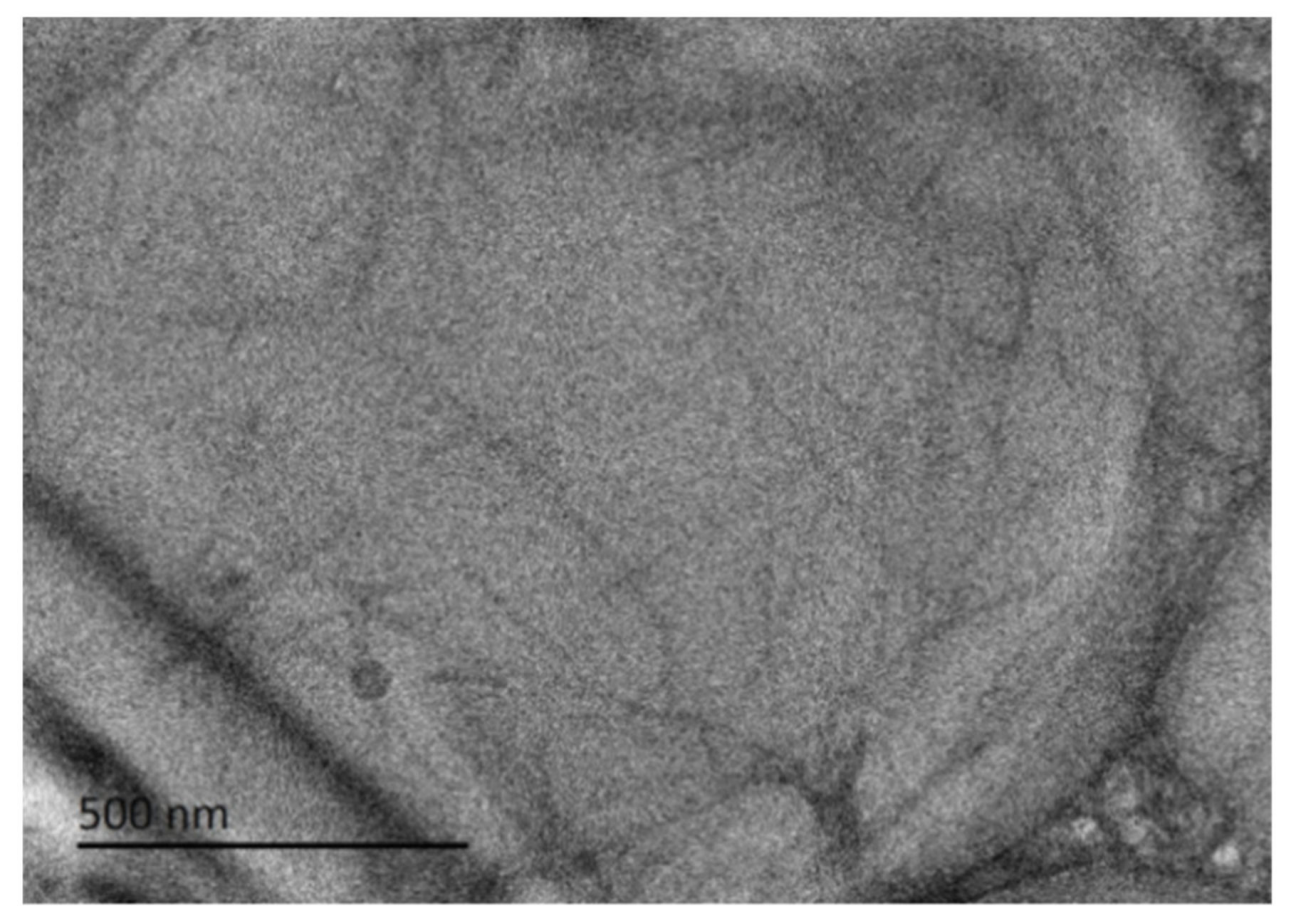
Cryo-TEM images of supramolecular hydrogel at 0.5 M.

As shown in Figure 4, the microstructure of the hydrogel at 0.5 M was studied by cryo-TEM, which clearly shows that the aggregates of the hydrogel are fibers, most of which are as large as 500 nm in length. Unlike other low-molecular-weight gelator molecules, The rosin ammonium molecule contains a hydrophobic structure with a tricyclic hydrogenated phenanthrene backbone, allowing a unique pattern in the fiber that prevents the direct insertion of long alkyl chains and maintains the fiber integrity. It may also contain a rigid skeletal structure of rosin, and the diameter of this gel fiber is relatively coarse.

### 2.5. The CD Spectrum of the Supramolecular Hydrogel

**Figure 5:**
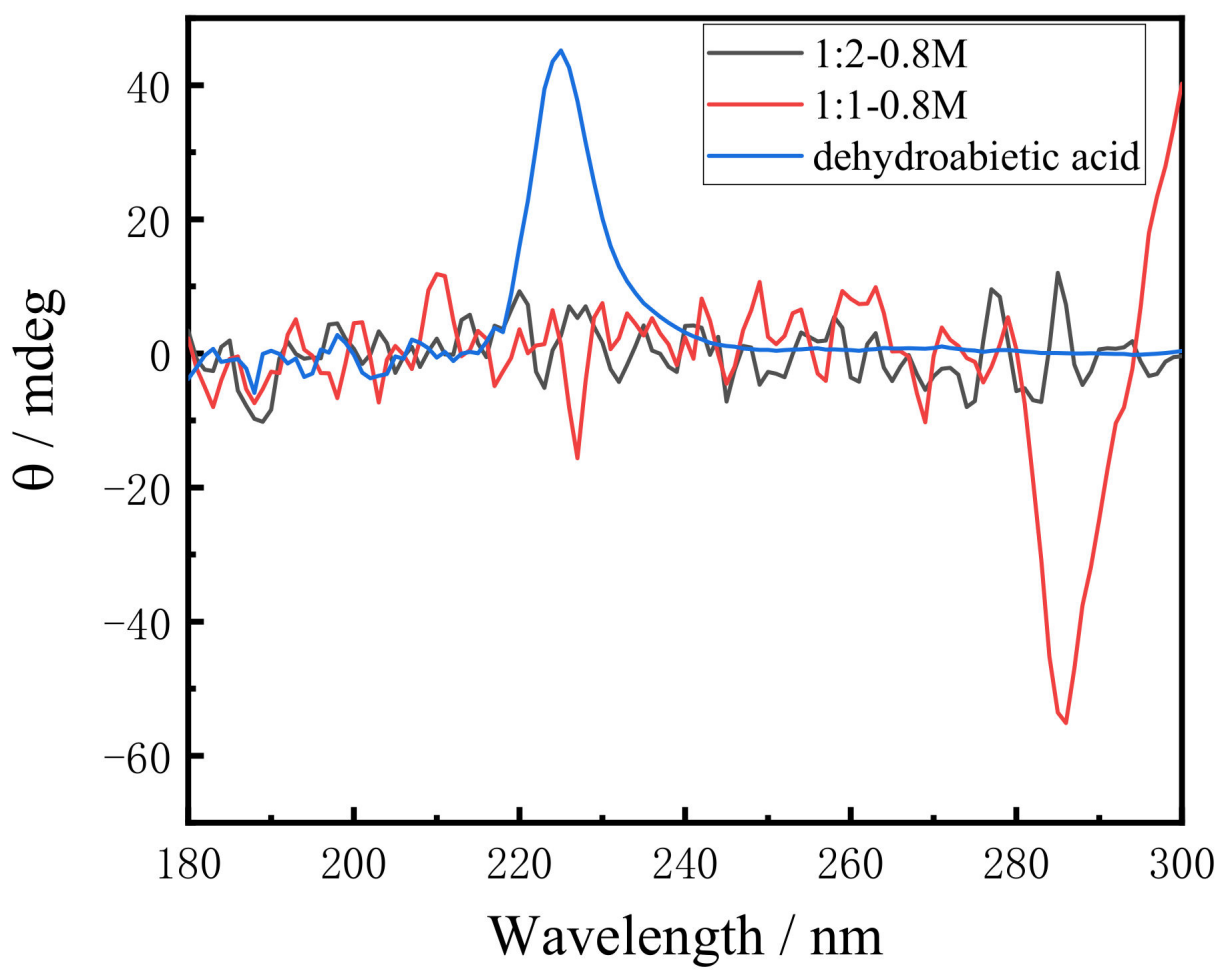
CD spectra of 1:1 and 1:2 system of dehydroabietic acid to morpholine and dehydroabietic acid.

In order to investigate the possible influence of the dehydroabietic backbone on molecular chirality, the CD spectrum of dehydroabietic acid was firstly measured. CD spectra are necessarily accompanied by artifacts due to macroscopic anisotropies such as linear birefringence (LB) and linear dichroism (LD), which are unique to the gel or solid state. The LB and LD signals of gels are negligibly small compared with CD, or if there are no macroscopic anisotropies-like solution samples, the signal observed is a true CD [45]. In the test analysis, the effect of LB and LD was so small that we ignored it and analyzed the CD spectrum in detail. As shown in Figure 5, the spectrum shows positive absorption peaks at 225 nm, which was led from the chiral carbon in the structure of dehydroabietic acid. On the other hand, the CD spectra at different molar ratios of dehydroabietic acid to morpholine show completely different CD signals. The hydrogel with a molar ratio of 1:1 shows a negative absorption peak at 225 nm and a large negative absorption peak at 285 nm. As the molar ratio increases, the negative absorption peak at 285 nm becomes a positive absorption peak when the molar ratio is 1:2. This is because a salt is formed during the reaction, which leads to a change in the rotation value and, eventually, a reversal of chirality. In this process, no chiral structures were generated in the synthesized gels and these chiral signals were mainly derived from the chirality of the dehydroabietic acid itself.

### 2.6. The Gelation Mechanism of Supramolecular Hydrogels

**Figure 6:**
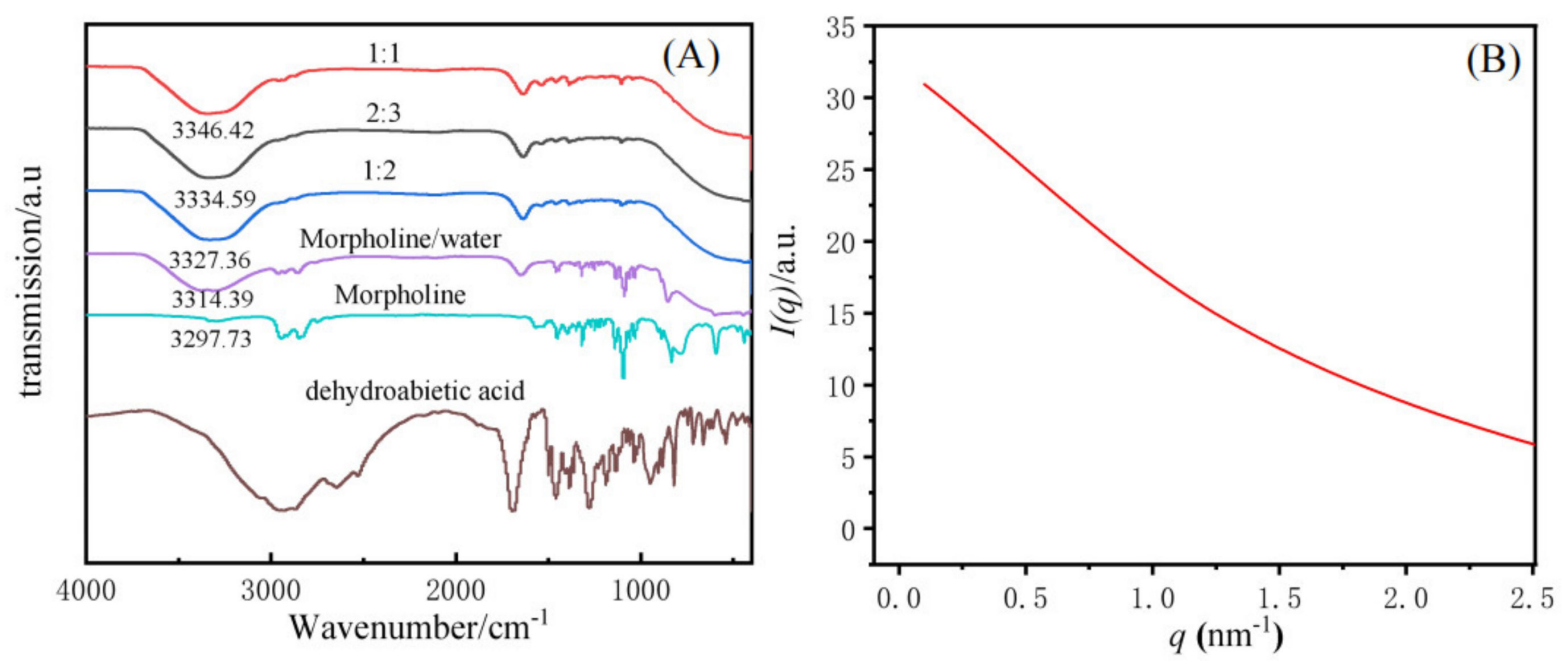
FT−IR picture of different molar ratio of the supramolecular hydrogels (**A**), SAXS spectrum of a 2:3 system of dehydroabietic acid–morpholine at 25 °C (**B**).

The gelation mechanism of dehydroabietic acid morpholine salt supramolecular hydrogels was investigated by FT-IR and SAXS. The IR spectra of the supramolecular gels with molar ratios of 1:1, 2:3, and 1:2, and raw materials with dehydroabietic acid, morpholine, morpholine/water were tested, respectively, and are shown in Figure 6A. The peak at 3297.73 cm^−1^ is the N-H peak of morpholine, while the peak at 3314.19 cm−1 is a broad peak formed by the N-H peak of morpholine and the O-H peak in water in the morpholine/water system. The comparison of the IR spectra of the hydrogels showed that the hydrogels with different molar ratios all had a broad peak at 3346.42 cm^−1^, 3334.59 cm^−1^, and 3327.36 cm^−1^, corresponding to molar ratios of 1:1, 2:3, and 1:2, respectively. Thus suggesting that the driving force for hydrogel formation is primarily the interaction of the N-H in morpholine with the hydrogen bonding of the O-H in water and -COOH in dehydroabietic acid. At the same time, because of the structure of the gel factor, Van der Waals forces and π-π stacking are also important factors in the formation of this supramolecular hydrogel.

SAXS was used to study the microscopic morphology of supramolecular hydrogels. Related literature reports that the aggregated structure of a substance can be inferred from the relationship between the scattering intensity and the scattering vector [46]. Figure 6B shows the scattering intensity (I) as a function of the scattering vector (q) in a 2:3 dehydroabietic acid to morpholine system at a concentration of 0.5 M. The SAXS curve shows a decay in intensity. In the range of low q, the intensity decreases with q, illustrating that the hydrogel is predominantly a cylindrical aggregated structure. The aggregated structure corresponds to the fiber structure tested by cryo-TEM. thicknesses could not be calculated directly using the Bragg equation [47,48,49]. Instead, the generalized indirect Fourier transformation (GIFT) method was adopted to determine the single wall thickness of the tubes [49,50]. The thickness to distance distribution function p_t_(r) profiles shown are observed to give a range of individual particle diameter distributions. It is not difficult to find that in the range below r = 5 nm, two-phase or multi-phase systems may exist, resulting in inhomogeneous particles with large and small sizes. Examples include particles with maximum diameters of 2.0 nm and 4.6 nm (Figure S5 in Supplementary Materials).

### 2.7. Preparation of the Supramolecular Gel Emulsions

**Figure 7:**
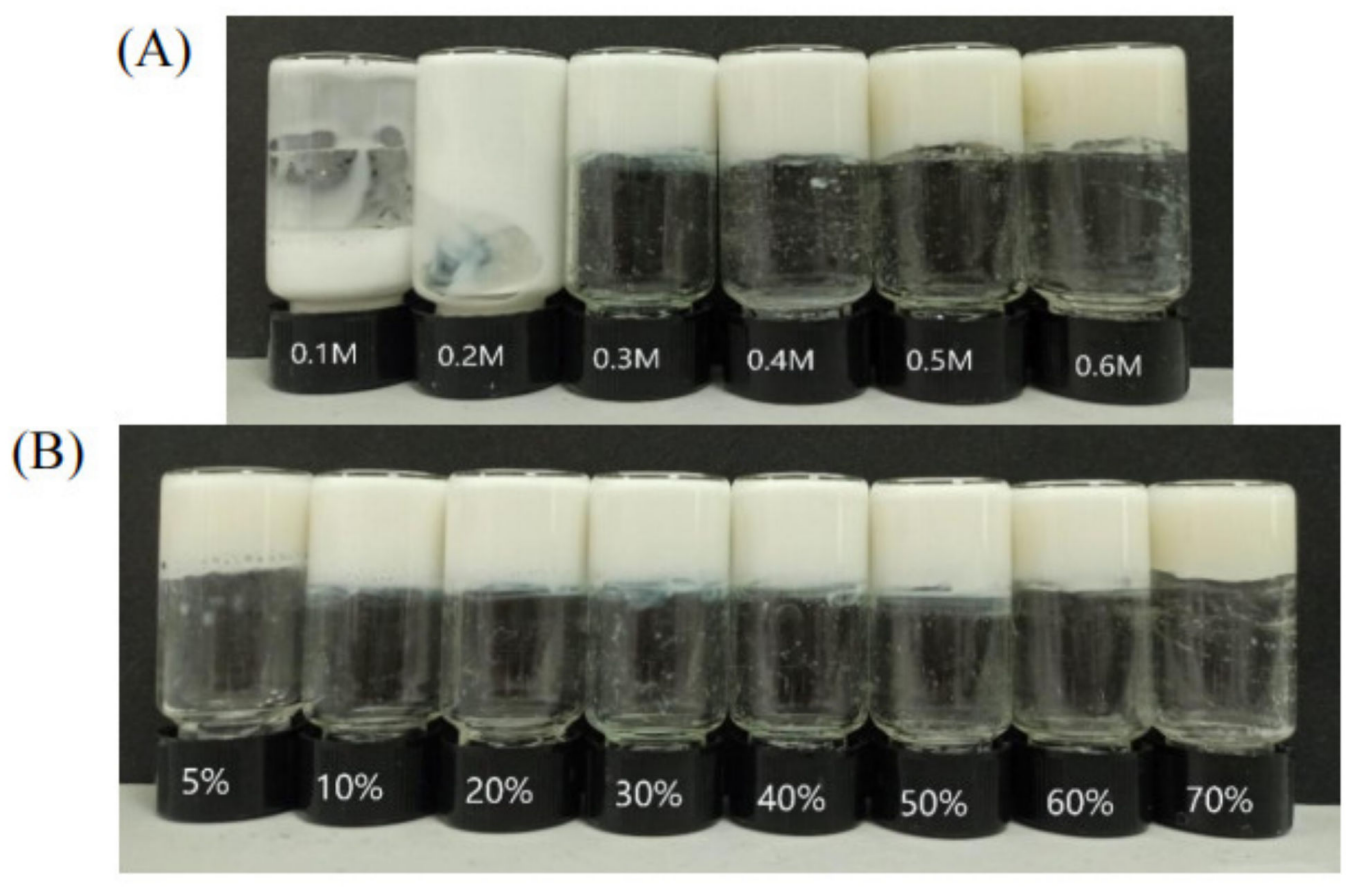
Digital photographs of n-hexadecane (50% oil) gel emulsions with different concentrations (**A**), Digital photograph of an aqueous n-hexadecane gel emulsion prepared at a constant concentration of 0.5 M but showing different oil mass fractions (**B**).

In order to research the emulsification properties of hydrogel agents, the gel emulsions of different mass ratios of oil to water were studied at a constant gelator concentration of 0.5 M. As shown in Figure 7A, gel emulsions with oil–water mass ratio of 50% and concentrations of 0.1,0.2, 0.3, 0.4, 0.5, and 0.6 M were prepared. It was found that when the concentration was greater than CGC, a stable water-in-oil gel emulsion could be formed and remained stable at 25 °C for several months, while the emulsion collapsed into a sol-gel emulsion when the gelling agent concentration was lower than 0.3 M. The stability of emulsions mainly comes from the continuous phase of the gel, which hinders the movement of dispersed droplets and thus prevents their flocculation and agglomeration. Moreover, as shown in Figure 7B, gel emulsions with different oil–water mass ratios of 5%, 10%, 20%, 30%, 40%, 50%, 60%, and 70% were prepared, and it was found that the volume fraction of oil in the emulsion could be freely chosen between 5% and 70%.

On the one hand, rosin ammonium molecules stabilize emulsions by adsorption at the oil–water interface through spatial stabilization such as common nonionic surfactants [1]. On the other handelf-assembly into fibers in water is responsible for aqueous phase gelation, which limits droplet motion and thus confers long-term stability to gel emulsions. 

### 2.8. CLSM of the Supramolecular Gel Emulsion

**Figure 8:**
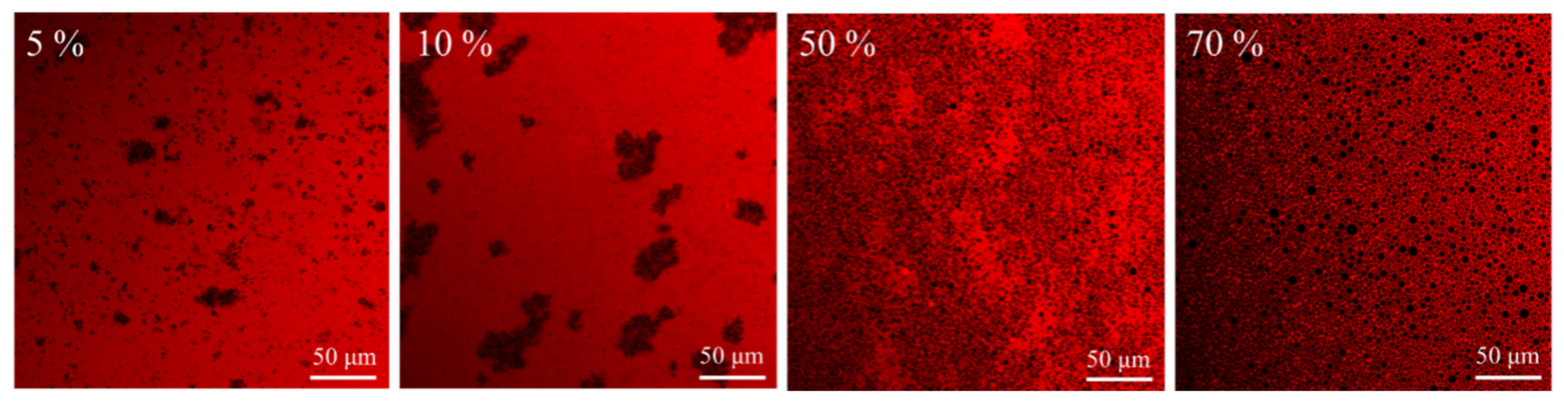
Confocal fluorescence images of n-hexadecane hydrogel emulsions with different oil mass fractions were stabilized by a concentration of 0.5 M gelator and taken after storage at 20 °C for 3 days.

The shape and size of the droplets in the gel emulsion were discovered by CLSM testing, as shown in Figure 8. It was discovered that at low oil mass fractions, the oil droplets are spherical and small in size but relatively aggregated in the continuous phase, and the size of the oil droplets increase with increasing oil mass fraction. These properties are outperformed by typical covalently bonded biopolymers used as aqueous thickeners, such as acacia bean gum, collagen, and carob gum, which need much higher concentrations to form gel emulsions [22,38,51].

## 3. Materials and Methods

### 3.1. Materials

Dehydroabietic acid (98% purity) was purchased from Hangzhou Wanjing New Materials Co. Morpholine (AR) and n-hexadecane were purchased from Aladdin (Shanghai, China). All other solvents and reagents are of analytical grade and can be used without further purification. 

### 3.2. Preparation of Rosin-Based Ammonium Salts

**Scheme 1:**
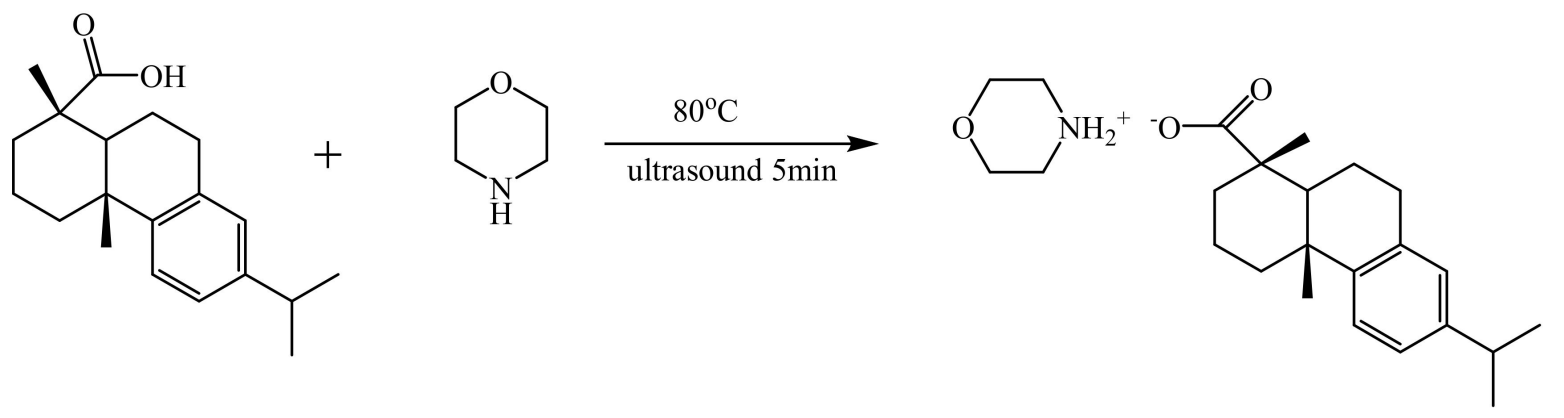
Synthesis of rosin−based ammonium salts.

A series of mixed systems of dehydroabietic acid and morpholine in different molar ratios and different concentrations were prepared by weighing them. Then deionized water was added and the mixture was heated to 80 °C and sonicated until all ingredients were completely dissolved. The synthesis of rosin-based ammonium salts were showed in Scheme 1. All gel samples were left to stand at 25 °C for at least 12 h before the relevant tests were carried out.

### 3.3. Preparation of Gel Emulsions

A certain amount of dehydroabietic acid and morpholine was added into a glass vessel of 10 mL, followed by adding pure water. The mixture was gently heated to 80 °C until dehydroabietic acid and morpholine was completely dissolved. Then desired amount of n-hexadecane was added. The mixture was kept at 80 °C for 0.5 h and was then homogenized using an Ultra-Turrax homogenizer (IKA T18 basic, DS-25 head) at 11,000 rpm for 2 min. The systems were cooled to 25 °C within 15 min to obtain different types of gel emulsions. 

### 3.4. Rheological Measurements

The rheological properties of this supramolecular hydrogel were performed with a rheometer (Discovery 100DHR-3). The diameter of the cone was 40 mm and the angle was 2°. Additionally, the gap between the center of the cone and the plate was 53 mm. During the whole experiment, the hydrogel samples were tested at 25 °C. More detailed test methods can be found in the literature [1].

### 3.5. Determination of the Gelation Temperature of Hydrogels

The gelation temperature of the hydrogels was determined by Differential Scanning Calorimeter DSC8000 (Perkin Elmer, Inc., USA). The temperature range was scanned at a rate of 5 °C/min, 30–150 °C under N2 atmosphere. The temperature of the samples was first increased to 150 °C and then cooled to 30 °C to eliminate any heat traces. After that, the samples were kept at 30 °C for 30 min before testing.

### 3.6. Small-Angle X-ray Scattering (SAXS)

The hydrogel was first heated to 80 °C and then injected into a capillary tube using a syringe. The sample was left to equilibrate at 25 °C for 12 h. SAXS measurements were carried out on a small-angle scatterometer (Anton-Paar SAX Ses mc2 system). The wavelength was 0.15406 nm. The voltage was 40 KV, the current was 30 mA and the point light source.

### 3.7. Cryogenic Transmission Electron Microscopy (cryo-TEM)

Using a pipette gun, 3 μL of the hydrogel was added to the TEM copper grid. The samples were then stored in liquid nitrogen, awaiting testing, and assayed with Talos F200C. More detailed test methods can be found in the literature [1].

### 3.8. Circular Dichroism Spectroscopy (CD)

The circular dichroism spectrum of the hydrogel was measured by using a MOS-450 spectrometer. The hydrogel was heated to 80 °C, and then the sample was injected into a quartz cuvette using a syringe. To ensure the formation of the hydrogel, the cuvette containing the sample was left to stand at 25 °C for 12 h. The tests were scanned from 180 to 300 nm wavelength.

### 3.9. Confocal Laser Scanning Microscopy (CLSM)

Confocal laser scanning microscopy experiments of this hydrogel were performed on a Leica TCS SP8 confocal microscope. The aqueous phase of the supramolecular hydrogel was stained using rhodamine B. The stained samples were placed on slides and then covered with coverslips for testing.

## 4. Conclusions

In conclusion, we have demonstrated a convenient strategy to construct a new low-molecular-weight supramolecular hydrogel by directly mixing two components and stable oil-in-water gel emulsions from hydrogel. The hydrogel can gel at 0.3 M and stable oil-in-water gel emulsions with oil mass fractions between 5 % and 70 %. Moreover, rosin is an FDA-approved food additive and morpholine is an FDA-approved fruits coating; the bio-based hydrogel meets the requirements of green development. It is possible that the good properties of rosin-derived rosin ammonium stable gel emulsions make them potentially useful in a wide range of applications such as the preparation of new porous materials, the transport of petroleum products, and in cosmetics and pharmaceuticals.

## Data Availability

The data presented in this study are available in Supplementary Material.

## Supplementary Materials

The following are available online, Figure S1: FT-IR picture of morpholine salt of dehydroabietic of the synthesized from dehydroabietic acid and morpholine; Figure S2: 1H-NMR (500 MHz) Spectra of morpholine salt of dehydroabietic in DMSO-d6; Figure S3: Appearance of hydrogel of morpholine salt of dehydroabietic of the synthesized from dehydroabietic acid and morpholine; Figure S4: DSC curve of hydrogel of different concentration at 25 °C (molar ratio of 1:1.5); Figure S5: (a) Normalized SAXS profile for the sample from dehydroabietic acid: morpholine at 25°C; (b) the corresponding p(r) profile.

## Sample Availability

Samples of the compounds are not available from the authors.
