# Supplementary material for: Facile Construction of Bio-Based Supramolecular Hydrogels from Dehydroabietic Acid with a Tricyclic Hydrophenanthrene Skeleton and Stabilized Gel Emulsions
Source: Molecules. 2021 Oct 28;26(21):6526. doi: 10.3390/molecules26216526 (PMC8586928; doi:10.3390/molecules26216526)
Supplement: Supplementary file 1 [file molecules-26-06526-s001.zip › molecules-1434104-supplementary.pdf]

# Supporting Information

## Facial construction of supramolecular hydrogels from dehydroabietic acid with a tricyclichydrophenanthrene skeleton and stabilized gel emulsions

Caiyun Lin, Yuying Li, Tang Weishan, Shufeng Zhou, Xiaoping Rao*

*College of Chemical Engineering, Huaqiao University, Xiamen, Fujian Province, China, 361021*

This supporting information contains 5 Figures.

1、Preparation of morpholine salt of dehydroabietic

Equimolar amounts of dehydroabietic acid and morpholine were loaded into a round bottom flask with a magnetic rotor, and an appropriate amount of ethyl acetate solvent was added and a reflux device was connected. The reaction was refluxed for 1 hour, and then the ethyl acetate was removed by rotary evaporator. It was recrystallized twice with ethanol and dried to obtain morpholine salt of dehydroabietic solid. Yield: 48.9%.

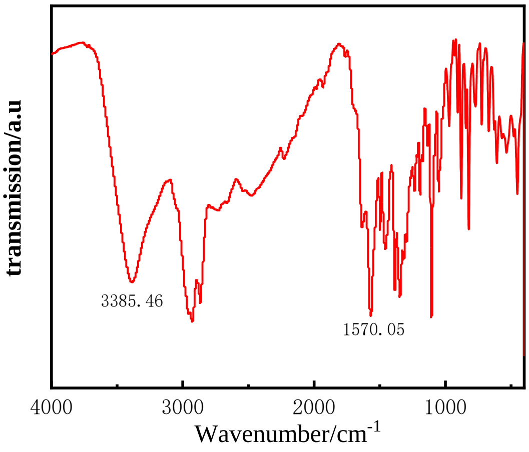


Fig. S1 FT-IR picture of morpholine salt of dehydroabietic of the synthesized from dehydroabietic acid and morpholine

the IR spectrum of the synthesized dehydroabietic morpholine salt was tested. The spectrum had a peak of N-H at 3385.46 $cm^{-1}$ and a peak at 1570.05 $cm^{-1}$ representing $-COO^{-}$. It indicates that the target morpholine salt of dehydroabietic was synthesized.

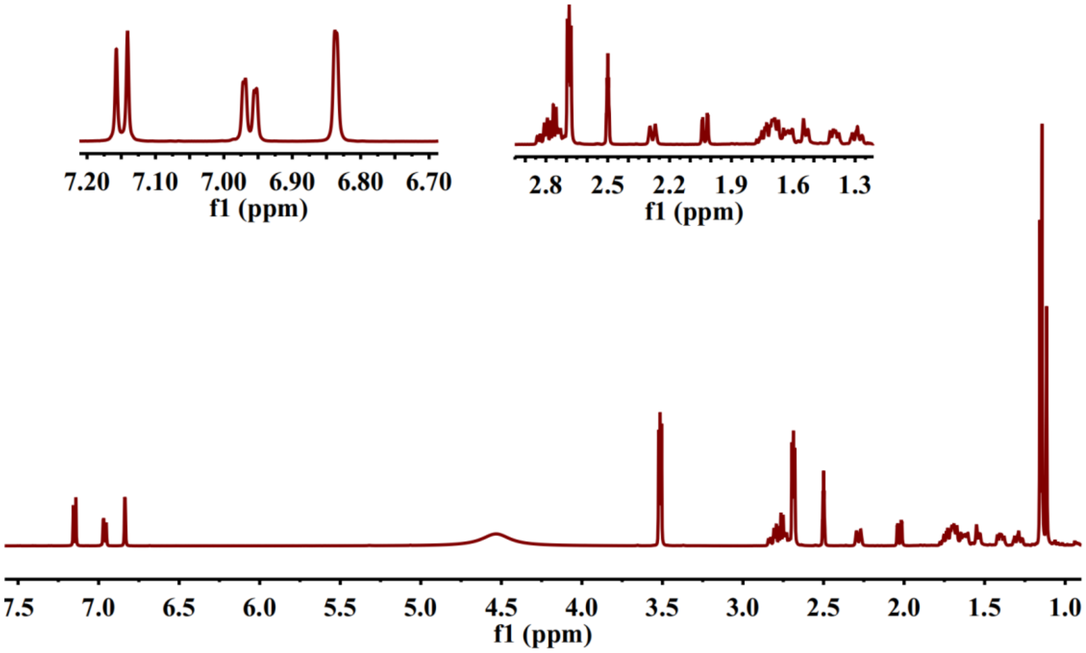


Fig. S2 1H NMR (500 MHz) Spectra of morpholine salt of dehydroabietic in DMSO-d6.

(500Hz,DMSO):7.15(d,2,10Hz,-$NH_2$),6.96(d,1,10Hz,-CH-),6.84(s,1,-CH-),3.52(t,4,15Hz,-$CH_2$-),2.76(m,2,50Hz,-$CH_2$-),2.84(s,1,-CH-),1.39(m,3,30Hz,-$CH_3$,),1.13(d,9,25Hz,-$CH_3$)

A gel can be formed by dissolving morpholine salt of dehydroabietic in an aqueous solvent and heating and sonicating. morpholine salt of dehydroabietic can also form a gel by adding the right amount of morpholine and heating and sonicating in aqueous solvent. However, the gel formed by first synthesizing morpholine salt of dehydroabietic and then heating and sonicating in aqueous solvent was not stable, and crystals would be precipitated after almost a day. This may be due to the addition of ethyl acetate organic solvent in the preparation process or ethanol solvent in the recrystallization process, which has an effect on the stability of the gel. Therefore, we prepared supramolecular hydrogels of morpholine salt of dehydroabietic, using a direct mixture of dehydroabietic and morpholine formed in heating and sonication.

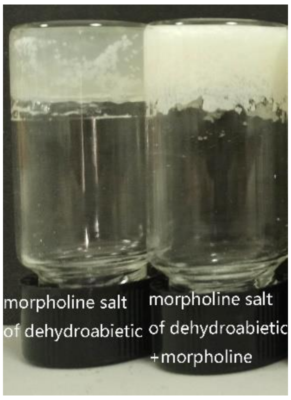


Fig. S3 Appearance of hydrogel of morpholine salt of dehydroabietic of the synthesized from dehydroabietic acid and morpholine

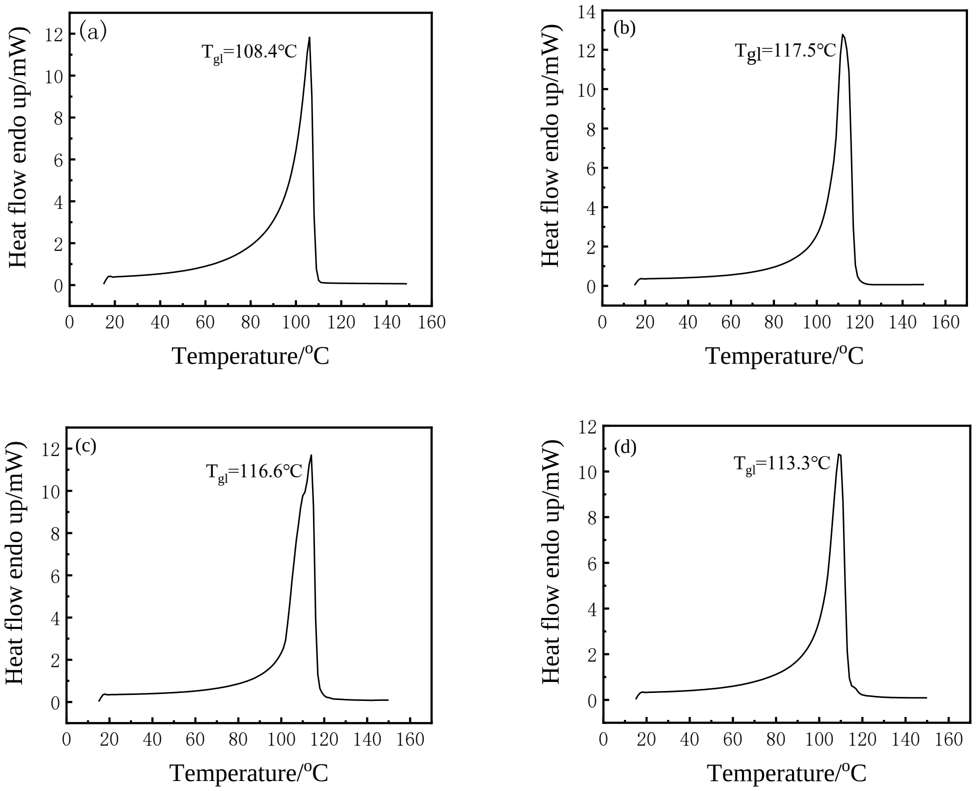


Fig. S4 DSC curve of hydrogel of different concentration at 25 °C (molar ratio of 1:1.5).

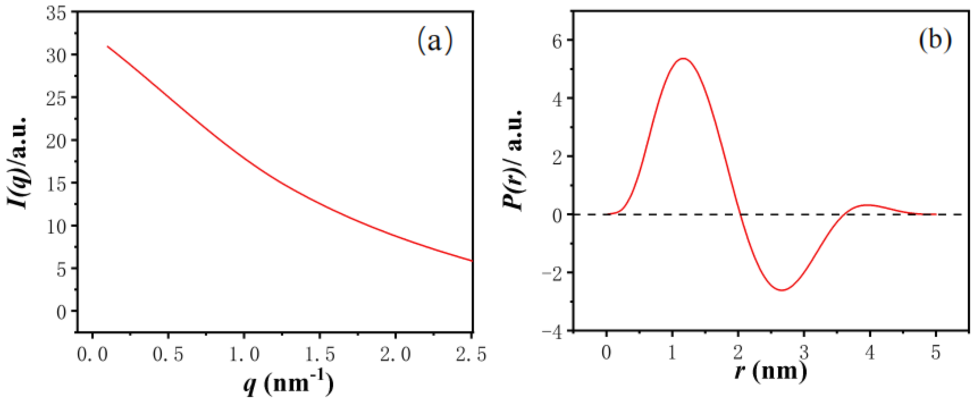


Fig. S5 (a) Normalized SAXS profile for the sample from dehydroabietic acid : morpholine at 25°C; (b) the corresponding p(r) profile.
